# Evaluation of Gait Smoothness in Patients with Stroke Undergoing Rehabilitation: Comparison between Two Metrics

**DOI:** 10.3390/ijerph192013440

**Published:** 2022-10-18

**Authors:** Marco Germanotta, Chiara Iacovelli, Irene Aprile

**Affiliations:** 1 IRCCS Fondazione Don Carlo Gnocchi, 50143 Florence, Italy; 2 Department of Aging, Neurological, Orthopaedic and Head-Neck Sciences, Fondazione Policlinico Universitario Agostino Gemelli IRCCS, Largo Francesco Vito 1, 00168 Rome, Italy; 3 Rehabilitation and Physical Medicine Unit, Fondazione Policlinico Universitario Agostino Gemelli IRCCS, Largo Francesco Vito 1, 00168 Rome, Italy

**Keywords:** smoothness, gait analysis, stroke, rehabilitation

## Abstract

The use of quantitative methods to analyze the loss in gait smoothness, an increase in movement intermittency which is a distinguishing hallmark of motor deficits in stroke patients, has gained considerable attention in recent years. In the literature, the spectral arc length (SPARC), as well as metrics based on the measurement of the jerk, such as the log dimensionless jerk (LDLJ), are currently employed to assess smoothness. However, the optimal measure for evaluating the smoothness of walking in stroke patients remains unknown. Here, we investigated the smoothness of the body’s center of mass (BCoM) trajectory during gait, using an optoelectronic system, in twenty-two subacute and eight chronic patients before and after a two-month rehabilitation program. The two measures were evaluated for their discriminant validity (ability to differentiate the smoothness of the BCoM trajectory calculated on the cycle of the affected and unaffected limb, and between subacute and chronic patients), validity (correlation with clinical scales), and responsiveness to the intervention. According to our findings, the LDLJ outperformed the SPARC in terms of the examined qualities. Based on data gathered using an optoelectronic system, we recommend using the LDLJ rather than the SPARC to investigate the gait smoothness of stroke patients.

## 1. Introduction

Mobility deficits are present in 70–80% of stroke survivors [1], often resulting in impaired gait, which persists despite rehabilitation [2], and negatively impacts physical activity [3]. Common gait problems include slowed walking, decreased stride length and cadence [4], and increased temporal asymmetry [5] which limit home and community ambulation and are associated with increased dependency and reduced quality of life [6]. Discovering treatments that address gait impairment, balance, and mobility is recognized as one of the highest priorities in post-stroke living [7]; nevertheless, quantitative methods that are reliable, clinically valid, and responsive to therapy are required to accurately measure their effectiveness.

Several investigations on upper limb impairment following stroke have shown that a decrease in movement smoothness, i.e., an increase in movement intermittency, is a defining characteristic of motor impairments [8,9]. Upper limb movement after a stroke seems to comprise a succession of distinct submovements [10], which may explain the observed lack of smoothness when compared with healthy subjects performing the same movements. In addition, it has been shown that, as a consequence of a rehabilitation intervention, these submovements eventually overlap and merge to produce a more fluid movement over time [11]. Several measures mostly based on the jerk (the third derivative of the displacement) have been proposed to assess smoothness. Among them, the log dimensionless jerk (LDLJ) [12] is considered the most valid, as it properly quantifies deviations from smooth, coordinated movements [13]. Moreover, a novel metric based on the arc length of the Fourier magnitude spectrum within an adjustable frequency range has been developed, namely, the spectral arc length measure (SPARC) [14].

Recently, in addition to upper limb movements, there has been an increase in interest in the use of quantitative methods to study gait smoothness. Some studies use Inertial Measurement Units (IMUs) placed on the lower back to quantify gait smoothness using the Harmonic Ratio index [15,16], despite this metric primarily reflecting gait asymmetry and not gait smoothness [17]. Few studies have analyzed the smoothness of gait using specific smoothness metrics, such as those previously mentioned, derived from the body’s center of mass (BCoM) trajectory estimated by optoelectronic systems, which are the gold standard for motion analysis, or IMUs. These studies provided an objective measurement of the gait smoothness in healthy subjects [18], athletes [19], geriatric subjects [20,21], or patients with Parkinson’s disease [17,21]. A study on young and older healthy subjects showed better performance of the LDLJ when compared with the SPARC, in identifying age-related stair negotiation adaptations and showed a better association with a clinical test of gait [20]. 

To the best of our knowledge, only one study has addressed the assessment of gait smoothness in stroke survivors, using the SPARC as a measure of smoothness [22], while no studies using jerk-based metrics to assess gait smoothness in stroke were identified. Then, a comparison between the two metrics was not performed in a cohort of patients with stroke and, consequently, it is unknown which measure should be used. 

Therefore, the purpose of this study is to quantify the ability of two metrics, namely, the LDLJ and the SPARC, to assess gait smoothness in a cohort of patients who have had a stroke by investigating: (a) their ability to differentiate between the BCoM trajectory during the gait cycle of the affected and unaffected limb, as well as between two groups of patients in a different stroke phase (subacute vs. chronic phase); (b) their correlation with clinical scales; and (c) their responsiveness to rehabilitation treatment.

## 2. Materials and Methods

### 2.1. Participant

We retrospectively analyzed gait analysis data of ambulatory patients with stroke admitted to our inpatient rehabilitation facility. This study was conducted in accordance with the Declaration of Helsinki, and retrospectively approved by the Ethics Committee “Comitato Etico Lazio 1” (protocol number 1334/CE Lazio 1, 5 November 2020). 

We included patients: (a) with an ischemic or hemorrhagic stroke confirmed by imaging (tomography or magnetic resonance imaging); (b) aged between 18 and 80 years; (c) able to walk at least 10 m with or without assistive devices; and (d) who performed at least two gait analysis tests, one upon admission and one after a two-month rehabilitation intervention. We excluded subjects with orthopedic diseases that could have limited the range of motion during gait. According to the inclusion and exclusion criteria, thirty patients were included in the research. The following demographic data have been extracted from the electronic medical records: age, sex, distance from the acute event, and clinical evaluation at the admission (T0) and discharge (T1). According to the time since stroke onset, patients were categorized as subacute patients (7 days–6 months), and chronic patients (>6 months) [23]. Based on this classification, 22 subjects were considered subacute patients, while 8 subjects were chronic patients.

The clinical evaluation was retrieved from clinical records and included the Functional Ambulatory Scale [24], the Timed-Up and Go test [25], the Six Minute Walk Test [26], and the Motricity Index [27] for the lower extremities. The interval between the clinical and the instrumental examinations did not exceed three days.

### 2.2. Rehabilitation Intervention

The rehabilitation intervention included daily conventional rehabilitation sessions, lasting 45 min, six times/week, focused on lower limbs, sitting and standing training, balance, and walking. The treatment included: muscle strengthening exercises; stretching of the lower limb, and static and dynamic exercises for the recovery of balance in the supine and standing positions using assistive devices; training gait exercises with parallel bars or in open spaces performed both with and without assistive devices; training to ascend and descend stairs; exercises to improve proprioception in the supine, sitting and standing positions, using a proprioceptive footboard; exercises to improve trunk control. The duration of the treatment was 2 months. This is the standard rehabilitation intervention for patients with stroke admitted to our rehabilitation facility.

### 2.3. Instrumental Evaluation

**Figure 1:**
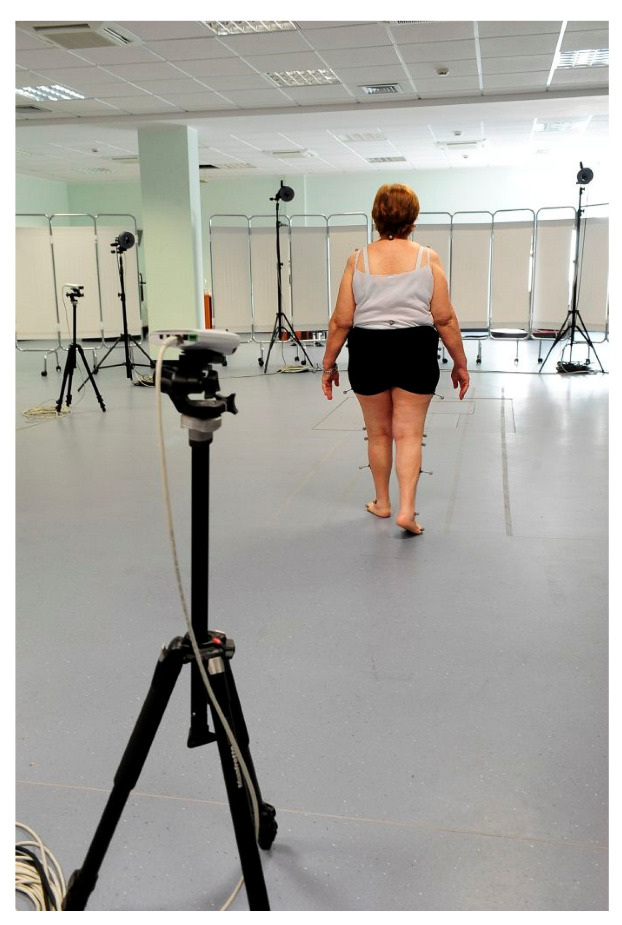
A participant performing a walking trial.

The instrumental evaluation was performed at the Movement Analysis Laboratory of the Fondazione Don Carlo Gnocchi, in Rome. It is equipped with an 8-camera stereophotogrammetric system (Smart D500, BTS Bioengineering, Milan, Italy). The calibrated volume of the SMART-D500 system was about (5 × 3 × 2) m^3^, within which the 3D coordinates of the retroreflective markers could be reconstructed with an accuracy of less than 1 mm in all directions, at a sampling rate of 200 Hz. The calibration of cameras was performed before each acquisition session, following the standard procedure described by the producer of the motion capture system. Patients were equipped with 22 retro-reflective markers, according to the Davis protocol [28]. Patients were then asked to perform barefoot overground straight walking trials (8.0 m walkway) at their natural speed (Figure 1). For each patient, at least 5 walking trials were analyzed.

### 2.4. Data Analysis

Data obtained through the stereophotogrammetric system were imported in MATLAB (version R2018a, MathWorks Inc., Natick, MA, USA). Specifically, markers’ trajectories were low-pass filtered (6 Hz cut-off, 5th order, zero-lag, Butterworth filter) and their gaps were filled through spline interpolation in case of short periods (maximum 10% of the entire stride) of markers’ masking. Markers placed on both feet were used to identify gait events, according to the algorithm proposed by O’Connor BCoM [29]. A visual inspection was performed on all the identified events, to ensure the accuracy of the method.

As previously suggested in the scientific literature [30], we approximated the BCoM as a fixed point in the center of the pelvis, defined as the centroid of the triangle generated by the two markers placed on the left and the right anterior superior iliac, and the one on the mid-point of the two posterior superior iliac spines. The BCoM displacement obtained through the stereophotogrammetric system was then numerically differentiated to obtain its velocity, acceleration, and jerk.

### 2.5. Smoothness Metrics

For each gait cycle, we computed the log dimensionless jerk (LDLJ) and the spectral arc length (SPARC), which are two validated smoothness metrics [14]. 

The LDLJ is defined as:

(1) $$LDLJ=-ln\left|\frac{{\left(t_{2}-t_{1}\right)}^{3}}{v_{peak}^{2}}\int_{t1}^{t2}{\left|\frac{d^{2}v\left(t\right)}{dt^{2}}\right|}^{2}dt\right|$$

where *v*(*t*) is the speed of the BCoM, *t* is the time, *t*_1_ and *t*_2_ are the start and end times of the movement (i.e., the two consecutive heel strikes of the analyzed cycle), and *v_peak_* is the peak speed of the BCoM.

The SPARC is defined as:

(2) $$SPARC=-\int_{0}^{\omega_{c}}{\left[{\left(\frac{1}{\omega_{c}}\right)}^{2}+{\left(\frac{d\hat{V}\left(\omega \right)}{d\omega }\right)}^{2}\right]}^{\frac{1}{2}}d\omega ;\hat{V}\left(\omega \right)=\frac{V\left(\omega \right)}{V\left(0\right)},$$

with:

(3) $$\omega_{c}≜\min\left\{\omega_{c}^{max},\min\left\{\omega ,\hat{V}\left(r\right)\langle \bar{V}\;\forall \;r\rangle \omega \right\}\right\}$$

where *V*(*ω*) is the Fourier magnitude spectrum of the speed *v*(*t*) of the BCoM, $\hat{V}\left(\omega \right)$ is the normalized magnitude spectrum, normalized to the DC magnitude *V*(0).

The LDLJ and SPARC for each right and left cycle were calculated for each evaluation session and referred to as affected and unaffected cycles, according to each patient.

### 2.6. Statistical Analysis

Descriptive and inferential statistical analyses were performed using IBM SPSS Statistics software (version 28, IBM Corp., Armonk, NY, USA). The distribution of each parameter was verified using the Shapiro–Wilk test; since data were not normally distributed, non-parametric tests were performed. Demographic and clinical data were compared between the two groups using the Mann–Whitney U test, or Fisher’s exact test, as appropriate. The score of the clinical scales obtained before and after the rehabilitation program were compared through the Wilcoxon Signed Rank Test. 

To assess the discriminant validity of the two metrics, data obtained at T0 from the gait cycles of the affected and the unaffected limb were compared by using the Wilcoxon Signed Rank Test; similarly, data obtained in the two groups of patients (subacute and chronic patients) were compared using the Mann–Whitney U test. To evaluate the validity, the correlations between the smoothness metrics and the clinical scales were assessed by means of the Spearman Rank Correlation Coefficients. Finally, to assess the responsiveness of the metrics, data obtained in the two sessions (T0 and T1) in the whole sample were compared through the Wilcoxon Signed Rank Test. Except for the first analysis, in which we consider data from the affected and the unaffected side separately, for each patient we averaged LDLJ and SPARC data from all the available cycles. For each analysis, a *p*-value lower than 0.05 was deemed significant. 

## 3. Results

The demographic and clinical characteristics of the enrolled sample are listed in Table 1. Comparing the two groups (subacute and chronic patients), we found, as expected, a statistically significant difference in the time since stroke (*p* < 0.001), with a mean time since the event equal to about three months in the subacute group and about thirty months in the chronic group. Moreover, we observed better lower extremity function, mobility, and lower fall risk in subacute patients, as showed by the lower time required to perform the TUG test (*p* < 0.001), as well as higher endurance, as indicated by the higher distance covered during the 6MWT (*p* = 0.049); in contrast, the two groups were comparable in terms of lower limb strength, as measured by the MI (*p* = 0.597).

After the treatment, patients improved on all the selected clinical scales, as shown in Table 2.

**Figure 2:**
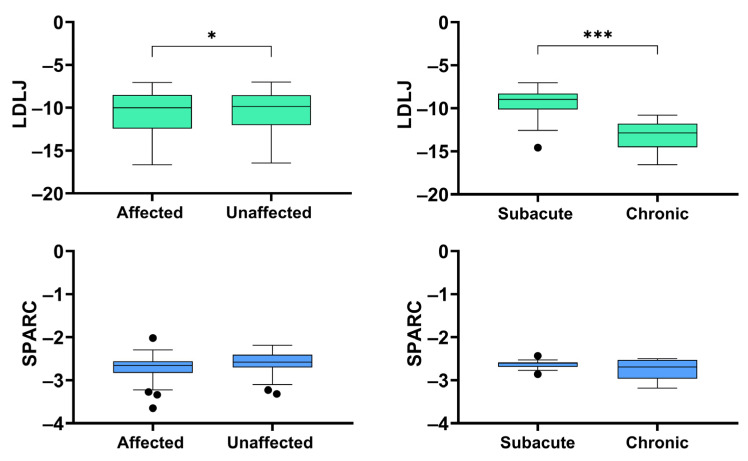
Discriminant validity analysis: ability of the two metrics (log dimensionless jerk, LDLJ, and spectral arc length, SPARC) in distinguishing the two gait cycles (affected or unaffected side), or subacute and chronic patients. The asterisks indicate a statistically significant difference (*: *p* < 0.05; *** *p* < 0.001).

Figure 2 reports the results related to the discriminant ability of the two metrics. Comparing data obtained from the BCoM kinematics during the gait cycle of the two sides, the Wilcoxon Sign Rank test revealed that the LDLJ was able to differentiate between them (*p* = 0.035), whereas the SPARC was not (*p* = 0.106). Similarly, according to the Mann–Whitney U test, the LDLJ discriminated between subacute and chronic patients (*p* < 0.001), while for the SPARC the difference between the two groups was not statistically significant (*p* = 0.298).

Table 3 reports the correlation analysis aimed to assess the validity of the two investigated metrics. The LDLJ was significantly and positively associated with the 6MWT and the MI and negatively correlated with the TUG, meaning that better clinical data were consistently associated with higher gait smoothness, as expected. Similarly, the SPARC was negatively associated with the TUG, and positively associated with the 6MWT; in contrast, it was not associated with the MI. Neither of the metrics was associated with the FAC. 

**Figure 3:**
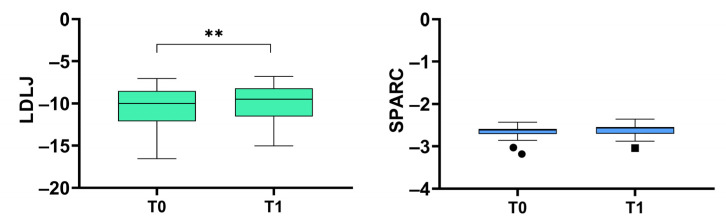
Responsiveness analysis: ability of the two metrics (log dimensionless jerk, LDLJ, and spectral arc length, SPARC) in following the clinical improvement after a rehabilitation intervention. The asterisks indicate a statistically significant difference (**: *p* < 0.01).

Figure 3 depicts the results of the responsiveness analysis. Comparing the LDLJ values obtained in the two evaluation sessions, we found a statistically significant increase in the metric, i.e., a higher smoothness (*p* = 0.005). A similar result was not obtained with the SPARC since the Wilcoxon test did not detect a statistically significant variation between the two evaluations (*p* = 0.221). 

## 4. Discussion

Smoothness, which refers to a subject’s capacity to produce continuous or non-intermittent movements [31], is a characteristic of voluntary healthy movements [32]. In this study, using kinematic data obtained from an optoelectronic system, the gold standard for motion analysis, we analyzed two metrics for assessing gait smoothness in two samples of patients with stroke (subacute and chronic patients) undergoing rehabilitation. Specifically, we compared the discriminant ability, the validity and the responsiveness of the log dimensionless jerk and the spectral arc length, obtained from the trajectory of the body’s center of mass.

Our results showed that the LDLJ satisfied all the analyzed psychometric properties, i.e., discriminant ability, validity and responsiveness. In fact, it was able to discriminate between the unaffected and affected gait cycle and patients with chronic stroke from those in a subacute phase. With respect to the first point, it is notable that the two cycles overlap; as a result, we expected smaller disparities between the BCoM movement smoothness assessed during each cycle; therefore, the obtained results highlight the sensitivity of the metrics. A more expected result was the ability to distinguish between subacute and chronic patients, which, in our sample, were characterized by different walking abilities, as indicated by the clinical scales. The high sensitivity of the LDLJ to assess gait smoothness is consistent with the findings of Kang et al. [18], who determine that the BCoM jerk, normalized to movement distance and stride time, could distinguish, in a group of healthy subjects, gait trials performed while experiencing different emotions. Moreover, in our study it was well correlated with the measured clinical scales, especially with the TUG; it is worth noting that a similar association between the TUG and the LDLJ measured on the BCoM trajectory during walking was found in the study by Dixon et al. [20]. Finally, the metric was responsive to the rehabilitation treatment: after the intervention, we observed a significant improvement in all the investigated clinical outcomes and, similarly, an increase in the LDLJ. Even though there are no data about the changes in gait smoothness in patients with stroke after a rehabilitation treatment, to the best of our knowledge, this was an expected result, considering the number of scientific papers that reported the responsiveness of kinematic measures of movement smoothness to upper limb rehabilitation in patients with stroke [8,33]. 

Concerning the SPARC, the statistical analysis confirmed the correlation with the TUG, already observed by Dixon et al. [20]; however, the metric did not exhibit satisfactory results either in terms of discriminant validity, or responsiveness. In fact, it was unable to distinguish between data related to the cycle of the affected or the unaffected side, or between subacute or stroke patients with different walking abilities; and, similarly, it was not able to reflect changes in gait smoothness induced by rehabilitation treatment. These results are partially in agreement with those obtained by do Vale Garcia et al. [22], based on data collected by means of an IMU. Specifically, the Authors compared the SPARC measured based on the yaw, roll and pitch component of the angular velocity during gait in three groups, namely, mild/moderate stroke patients, severe stroke patients, and a control group, and only the SPARC based on the roll component of the angular velocity was able to discriminate stroke patients from the control group, but not between patients with different severity. However, we cannot directly compare these results with ours, since they investigated only chronic stroke patients, using different sensors to compute the SPARC. In contrast, better results were obtained in other studies that investigated the properties of the SPARC to quantify gait smoothness [17,21,34] in patients with Parkinson’s Disease (PD), based on data collected employing an IMU. Specifically, according to Beck et al. [17] the SPARC, measured during a walking task, was sensitive to the presence of the disease (i.e., able to distinguish patients from controls) and to PD medications (i.e., able to distinguish patients with and without anti-PD medication), as well as clinical valid, being correlated with the UPDRS motor score; similar results were obtained by Pinto et al. [21] during a functional mobility task (i.e., the TUG test). 

In the literature, only Dixon et al. [20] directly compared the properties of the LDLJ and the SPARC metrics in assessing BCoM smoothness during gait. Specifically, the authors used an optoelectronic system to measure BCoM and head kinematics in three different tasks (overground straight walking, stair ascent, and stair descent) in two cohorts of healthy subjects (older and young adults). Their results are consistent with ours since, according to their research, the LDLJ performs better than the SPARC as a smoothness metric for differentiating between the analyzed populations and tasks; in addition, the LDLJ demonstrated a meaningful negative correlation with the TUG test (i.e., an increase in smoothness corresponded to a decrease in the time required to complete the task), whereas a counterintuitive positive correlation was found between the TUG and the SPARC. Therefore, our results corroborate what was previously found in healthy subjects, suggesting that the LDLJ is preferable as a gait smoothness metric also in stroke patients, at least when using data provided by an optoelectronic system. 

According to the above, the Literature seems to suggest that different metrics could be more suitable for different movements or patients. Indeed, in a study by Caronni et al. [32], the authors investigated differences in the smoothness of head movements between healthy subjects and patients with idiopathic cervical dystonia, using the same indices here proposed, but obtaining opposite results: in fact, in their study, only the SPARC was able to distinguish between healthy subject and patient with cervical dystonia. The authors interpreted the better behavior of the SPARC in light of the study by Singh et al. [35], which suggested that the SPARC is more sensitive to changes in smaller movements. Given that our protocol involved large movements, this interpretation may also help to explain our findings. In addition, it is possible that linear motions (as in our study) and rotational movements (as in the study by Caronni et al. [32]) may require different metrics to evaluate smoothness. This corroborates the hypothesis that a better metric does not exist, but it should be selected according to the analyzed movement, as well as, the investigated cohort and the employed sensors to acquire the BCoM kinematics.

We should acknowledge some limitations of our study: the retrospective nature of the study; the reduced sample size; and, finally, the lack of a second evaluation session to analyze the reliability of the two metrics (test–retest reliability). Nevertheless, our results suggest that, in patients with stroke, the LDLJ is preferable to the SPARC as a measure of smoothness of the BCoM trajectory, measured with an optoelectronic system. Future studies should be addressed to compare the two metrics using data provided by wearable systems.

## 5. Conclusions

In this study, we examined the discriminant ability, validity, and responsiveness of two widely used metrics for assessing smoothness: the LDLJ and the SPARC. Using an optoelectronic system, we assessed the smoothness of the trajectory of the body’s Center of Mass during gait in two groups of patients with stroke. According to our findings, the LDLJ is preferable to the SPARC for assessing the gait smoothness of stroke patients using data provided by an optoelectronic system.

## Figures and Tables

**Table 1:** Demographic and clinical characteristics of the sample. *p* values refer to the Mann–Whitney U test, or Fisher’s exact test, as appropriate. Values in bold indicate a statistically significant difference between subacute and chronic stroke patients (*p* < 0.05).

Variable	Subacute (N = 22)	Chronic (N = 8)	*p*-Value
Age	63.1 (9.4)	69.1 (11.2)	0.185
Time since stroke (days)	99.9 (60.4)	911.3 (685.6)	**<0.001**
Sex (male)	18 (81.8%)	4 (50%)	0.158
Affected side (right)	9 (40.9%)	4 (50%)	0.698
Functional Ambulation Category	2.9 (1.6)	3.1 (1.8)	0.696
Timed Up and Go (s)	24.4 (13.2)	66.7 (58.4)	**0.013**
Six-Minute Walking Test (m)	201.2 (133.7)	106.7 (56.1)	**0.049**
Motricity Index	68.4 (19.8)	59.9 (30.8)	0.597

**Table 2:** Clinical assessment before (T0) and after the rehabilitation treatment (T1) for the two investigated groups. *p*-values refer to the Wilcoxon Sign Rank Test. Values in bold indicate a statistically significant difference between T0 and T1 (*p* < 0.05).

Variable	Subacute (N = 22)	Chronic (N = 8)	*p*-Value
Functional Ambulation Category	3.0 (1.6)	3.9 (1.1)	**<0.001**
Timed Up and Go (s)	36.1 (36.7)	33.5 (37.1)	**<0.001**
Six-Minute Walking Test (m)	174.2 (123.6)	203.8 (132.3)	**0.002**
Motricity Index	66.1 (22.9)	75.6 (23.3)	**<0.001**

**Table 3:** Spearman’s rank correlation coefficient (Spearman’s rho) and *p*-values between the clinical scales and the two metrics of smoothness (log dimensionless jerk, LDLJ, and spectral arc length, SPARC). Values in bold indicate a statistically significant correlation (*p* < 0.05).

Variable	LDLJ Spearman’s Rho (*p*)	SPARC Spearman’s Rho (*p*)
Functional Ambulation Category	0.309 (0.097)	0.163 (0.390)
Timed Up and Go (s)	**−0.808 (<0.001)**	**−0.465 (0.011)**
Six-Minute Walking Test (m)	**0.741 (<0.001)**	**0.433 (0.021)**
Motricity Index	**0.438 (<0.001)**	0.307 (0.099)

## Data Availability

Data are available from the corresponding author upon reasonable request.
